# Case report: Sustained remission after combined sintilimab, anti-VEGF therapy, and chemotherapy in a patient with non-small cell lung cancer harboring acquired *EGFR* 19Del/T790M/*cis*-C797S mutation resistance

**DOI:** 10.3389/fonc.2024.1298389

**Published:** 2024-06-06

**Authors:** Wanming He, Lihua Tong, Wen Yang, Yanling Yuan, Yu Li, Wubing Tang

**Affiliations:** Department of Oncology, The Sixth Affiliated Hospital, School of Medicine, South China University of Technology, Foshan, China

**Keywords:** non-small cell lung cancer, growth factor receptor, tyrosine kinase inhibitors, programmed cell death 1 inhibitor, anti-vascular endothelial growth factor therapy

## Abstract

Third-generation epidermal growth factor receptor (EGFR) tyrosine kinase inhibitors (TKIs) are highly effective against tumors harboring the T790M mutation. However, patients treated with these inhibitors ultimately develop resistance, and the most common mechanism is the emergence of the *EGFR* C797S mutation. Few treatment regimens have been reported for this condition. In this report, we present a successful combination treatment with the programmed cell death 1 (PD-1) inhibitor sintilimab, anti-vascular endothelial growth factor (VEGF) therapy, and chemotherapy with pemetrexed and cisplatin in a patient with non-small cell lung cancer (NSCLC) who developed acquired resistance with *EGFR* 19 exon deletion (19Del)/T790M/*cis-*C797S mutation following progression with ametinib therapy. This regimen was well tolerated, and the patient has remained progression-free for 15 months. Our case provides clinical evidence that the combination of PD-1 inhibitor, anti-VEGF therapy, and chemotherapy may be an efficacious therapeutic strategy for NSCLC patients with acquired *EGFR* 19Del/T790M/*cis-*C797S mutation resistance following progression with EGFR TKI therapy.

## Introduction

Despite initial response to epidermal growth factor receptor (EGFR) tyrosine kinase inhibitors (TKIs), most patients with non-small cell lung cancer (NSCLC) harboring *EGFR* activating mutations inevitably develop resistance after approximately one year (1, 2). The *EGFR* T790M mutation is the most common mechanism of resistance to first- and second-generation EGFR TKIs, and third-generation EGFR TKIs, such as osimertinib and ametinib, selectively target the T790M mutation. However, patients treated with third-generation EGFR TKIs ultimately encounter secondary resistance. Although the mechanisms of resistance vary, the most common is the emergence of the *EGFR* C797S mutation (3), with reported frequencies up to 24% (4–6). According to the allelic relationship with T790M, C797S is defined as *cis*-C797S or *trans*-C797S (7). Tumors harboring T790M/*trans*-C797S are sensitive to combined first- and third-generation EGFR TKIs (7, 8). However T790M/*cis*-C797S, the more frequently mutation, is resistant to first-, second-, and third-generation EGFR TKIs (3, 9). Currently, there is no standard therapeutic regimen for NSCLCs harboring the T790M/*cis*-C797S *EGFR* mutation. Platinum-based chemotherapy with or without bevacizumab is one of the recommended regiments (10), however, the survival is poor. Here, we report a successful case of combination therapy with PD-1 inhibitor (sintilimab), anti-VEGF therapy, and chemotherapy in a patient with NSCLC who developed acquired *EGFR* 19 exon deletion (19Del)/T790M/*cis*-C797S mutation resistance following progression on EGFR TKI therapy.

## Case report

**Figure 1:**
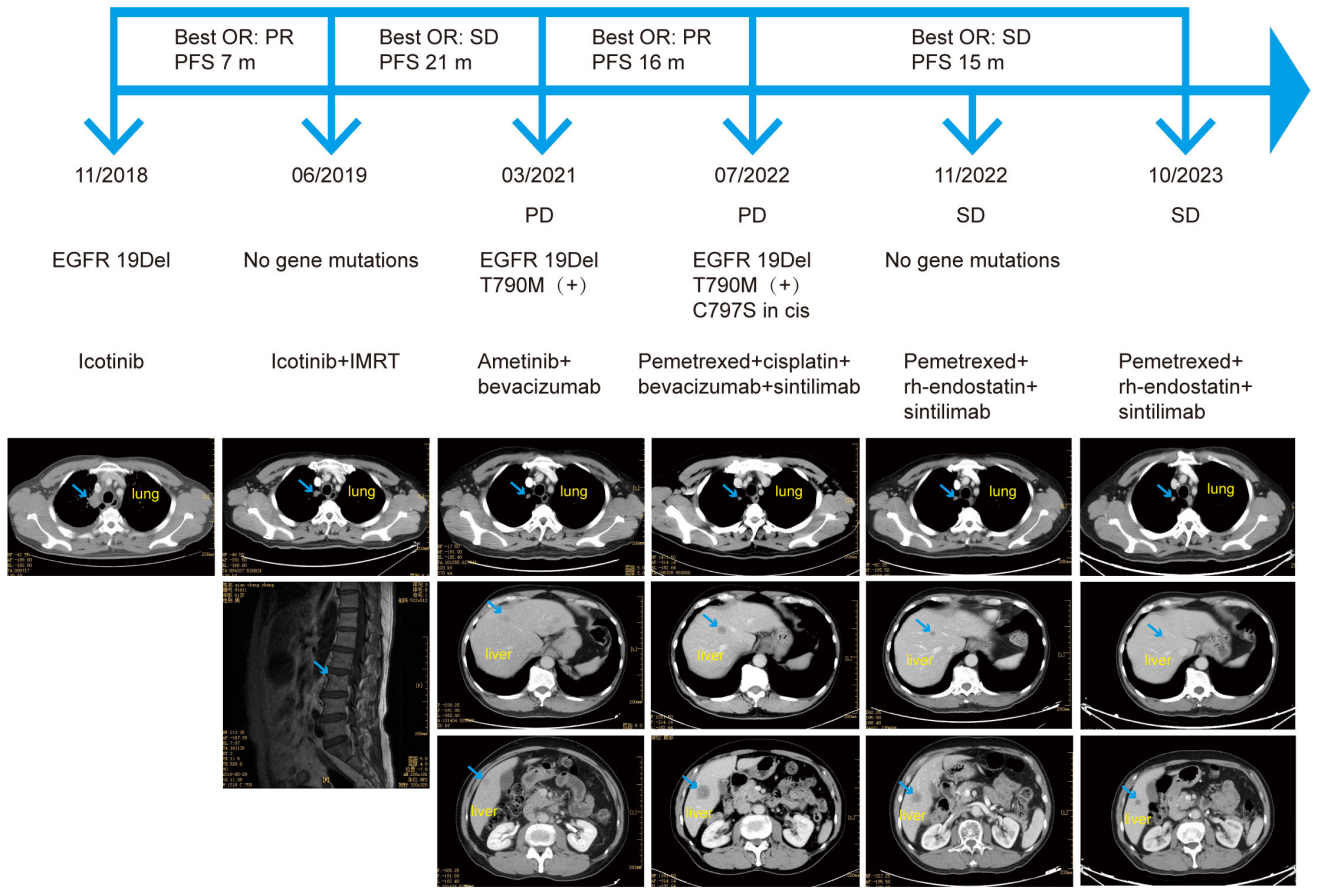
The patient’s course, treatment, next-generation sequencing results, and imaging results. OR, objective response; PFS, progression-free survival; PR, partial response; SD, stable disease; IMRT, intensity-modulated radiotherapy; *EGFR*, epidermal growth factor receptor; 19Del, exon 19 deletion; PD, progressive disease.

A 61-year-old man, a former smoker with no relevant family or genetic history, underwent computed tomography (CT) of the chest in November 2018, due to a cough. The CT scan revealed a nodule in the right upper lung near the mediastinum, suggesting a neoplastic lesion (**Figure 1**). One month later, he was diagnosed with Stage IVA (T4N2M1a) lung adenocarcinoma with brain metastasis in the left occipital lobe. Genomic profiling of pleural effusion cell pellets using next-generation sequencing (NGS) identified an *EGFR* 19 exon delete (19Del; c.2235_2249del p.Glu746_Ala750del). Consequently, he was treated with icotinib (125 mg tid), achieving a partial response (PR).

In June 2019, magnetic resonance imaging (MRI) revealed bone metastases at the L3 lumbar and S2 and S3 sacral vertebrae. He received intensity-modulated radiotherapy using RAPID-Arc, delivering 55 Gy in 22 fractions to gross target volume (GTV) and 40 Gy in 22 fractions to clinical target volume (CTV). Since bone-related examinations were not performed at the initial diagnosis, baseline images were unavailable. NGS analysis of a blood sample did not detect an *EGFR* mutation, and CT scans showed reduced lung lesions, indicating effectiveness of icotinib. Consequently, icotinib treatment was continued.

The patient maintained stable disease (SD) for 21 months, until CT scans revealed new lesions in both lungs and the liver. NGS analysis of a blood sample identified an *EGFR* T790M mutation (c.2369C>Tp.Thr790Met) along with the *EGFR* 19Del (c.2235_2249del p.Glu746_Ala750del). Subsequently, he commenced treatment with ametinib (110 mg, qd) combined with bevacizumab (400 mg q3w), achieving a PR. However, disease progression was observed in July 2022 with enlarged liver metastases and an increased number of liver lesions. NGS analysis of a blood sample revealed a novel *EGFR cis*-C797S mutation (c.2389T>Ap.Cys797Ser) in addition to the existing *EGFR* 19Del and T790M mutations.

The ORIENT-31 trial, a prospective, double-blind, phase 3 clinical trial, evaluated the efficacy and safety of sintilimab with or without bevacizumab biosimilar IBI305 plus pemetrexed and cisplatin, compared with pemetrexed and cisplatin alone, for patients with locally advanced or metastatic *EGFR*-mutated NSCLC who had disease progression after receiving EGFR TKI therapy (11). Based on the preliminary results from this trial, we initiated a treatment regimen of pemetrexed and cisplatin combined with bevacizumab and sintilimab (200 mg q3w) in July 2022 for our patient, who had an Eastern Cooperative Oncology Group performance status (ECOG PS) score of 1. After six courses of this regimen, he transitioned to maintenance therapy with pemetrexed, bevacizumab and sintilimab.

A CT scan in November 2022 showed that the primary lung lesion and multiple lung metastases were mostly unchanged, although the liver lesions had shrunk, indicating an objective response (OR) of SD. NGS analysis of a blood sample did not identify the *EGFR* 19Del, T790M, or *cis*-C797S mutations, and no other mutations were detected. Due to the patient’s worsening economic situation, bevacizumab was replaced with the lower cost recombinant human endostatin (30 mg, d1–7, q3w). As of October 2023, the patient continued to respond to the treatment regimen of pemetrexed combined with recombinant human endostatin and sintilimab, with a progression-free survival (PFS) exceeding 15 months. The only treatment-related side effect was grade 2 diarrhea, according to the Common Terminology Criteria for Adverse Events (CTCAE) Version 5.0, which occurred after four courses, and was alleviated with symptomatic treatment. A colonoscopy in November 2022 indicated no abnormalities.

## Discussion

Due to the molecular heterogeneity of NSCLC, the resistance mechanisms to third-generation TKIs are complicated and not fully understood. Acquired resistance to EGFR TKIs can be broadly categorized into *EGFR*-dependent (on-target) and *EGFR*-independent (off-target) (12, 13). Relevant therapeutic options have been found to prolong clinical benefits. For instance, the combination of the ALK inhibitor brigatinib with cetuximab may be effective for patients with acquired *EGFR* T790M/*cis*-C797S-mediated resistance to osimertinib (14, 15). Fourth-generation EGFR TKIs, such as EAI045, JBJ-04–125-02, and BLU-945, can overcome both the T790M and C797S mutations (16). Additionally, the phase III MARIPOSA-2 study demonstrated that PFS was significantly longer for the combination of amivantamabe-lazertinibe and chemotherapy compared to chemotherapy alone in patients with *EGFR*-mutated advanced NSCLC who had progressed on or after osimertinib (median of 8.3 versus 4.2 months, respectively) (17). Furthermore, the antibody-drug conjugate (ADC) patritumab deruxtecan (HER3-DXd) showed clinically meaningful efficacy in the phase II HERTHENA-Lung01 study, and a phase III HERTHENA-Lung02 trial is ongoing (ClinicalTrials.gov identifier: NCT05338970) (18).

In our case, the patient acquired an *EGFR cis*-C797S mutation after treatment with a third-generation TKI. However, fourth-generation EGFR TKIs are not readily accessible to Chinese patients in clinical practice, and the cost of brigatinib and cetuximab is high, increasing the financial burden on patients. Therefore, economical, accessible and effective therapeutic regimens are needed to manage those NSCLC Chinese patients who acquire an *EGFR cis*-C797S mutation.

A few randomized phase 3 trials have shown that combining PD-1 or programmed cell death ligand 1 (PD-L1) inhibitors with VEGF inhibitors and chemotherapy enhances antitumor activity and provides a PFS benefit for patients with advanced *EGFR*-mutated NSCLC who progressed after receiving EGFR TKI therapy. A subgroup analysis of the IMpower150 trial showed that treatment with the PD-L1 inhibitor atezolizumab, bevacizumab, and chemotherapy (carboplatin and paclitaxel) improved survival outcomes in NSCLC patients who developed *EGFR* mutations after TKI treatment (19, 20). Additionally, the ORIENT-31 trial demonstrated that treatment with the PD-1 inhibitor sintilimab, bevacizumab biosimilar IBI305, and standard chemotherapy (pemetrexed and cisplatin) significantly improved PFS compared to chemotherapy alone (median 7.2 months *vs* 4.3 months; hazard ratio 0.51; p<0.0001) for NSCLC patients who had progressed after EGFR TKI therapy (11). However, the trial included patients with multiple *EGFR* mutations, including exon 19Del, exon 21 L858R, and others, not exclusively those with acquired *EGFR cis-*C797S mutations. As of October 2023, the last follow-up time, our patient is still responding to the combination of a PD-1 inhibitor, anti-VEGF therapy and chemotherapy, with a progression-free survival (PFS) of over 15 months, exceeding the median PFS of 7.2 months reported in the ORIENT-31 trial.

To date, the mechanism of this treatment regimen remains unclear. Due to low response rates to immune checkpoint inhibitors (ICIs) in patients with *EGFR*-mutant NSCLC (21), this population has typically been excluded from first-line treatment with immunotherapy. Nevertheless, recent translational studies have shown that ICIs are more effective in patients with PD-L1 higher expression in tumor cells, a higher tumor mutation burden, or a higher density of tumor-infiltrating lymphocytes following EGFR TKI treatment (22–24). Moreover, multiple clinical studies have indicated that the efficacy of ICIs may be enhanced when combined with VEGF inhibitors (25–27). Anti-angiogenic therapy induces normalization of tumor vasculature, promoting T cell infiltration into the tumor and creating a tumor immune microenvironment favorable for ICI therapy (28, 29). Additionally, VEGF expression can be promoted by *EGFR* signaling, potentially increasing the sensitivity of tumors harboring *EGFR* mutations to anti-VEGF therapy (30, 31).

In our case, several limitations should be considered. Repeated tissue biopsies are necessary to identify histological changes in complex cancers and to elucidate resistance mechanisms if the combination treatment of a PD-1 inhibitor, anti-VEGF therapy, and chemotherapy fails. After three months of combination therapy, no gene mutations were detected, yet the patient continued to respond to treatment. The underlying mechanism warrants further investigation. Despite these limitations, the patient has acquired survival benefits and the three-drug regimen has been well tolerated. The only side effect was grade 2 diarrhea, which was alleviated with symptomatic treatment. Our case may shed lights on overcoming *EGFR* 19Del/T790M/*cis-*C797S mutation resistance.

## Conclusion

The combination treatment with the PD-1 inhibitor sintilimab, anti-VEGF therapy, and chemotherapy demonstrated a significant improvement in PFS in a NSCLC patient who developed acquired resistance due to *EGFR* 19Del/T790M/*cis-*C797S mutation after progression on EGFR TKI therapy. This therapeutic regimen may be efficacious and offers an optimal strategy for managing these patients.

## Data availability statement

The original contributions presented in the study are included in the article/**Supplementary Material**. Further inquiries can be directed to the corresponding author.

## Ethics statement

The studies involving humans were approved by The Clinical Research Ethics Committee of the Sixth Affiliated Hospital of South China University of Technology. The studies were conducted in accordance with the local legislation and institutional requirements. The participants provided their written informed consent to participate in this study. Written informed consent was obtained from the individual(s) for the publication of any potentially identifiable images or data included in this article.

## Author contributions

WH: Project administration, Software, Writing – original draft. LT: Investigation, Writing – review & editing. WY: Methodology, Writing – review & editing. YY: Writing – review & editing, Project administration. YL: Project administration, Writing – review & editing. WT: Funding acquisition, Supervision, Writing – review & editing.

## References

[1] Zhou C Wu Y Chen G Feng J Liu X Wang C . Erlotinib versus chemotherapy as first-line treatment for patients with advanced EGFR mutation-positive non-small-cell lung cancer (OPTIMAL, CTONG-0802): a multicentre, open-label, randomised, phase 3 study. Lancet Oncol. (2011) 12(8):735–42. doi: 10.1016/S1470-2045(11)70184-X 21783417

[2] Mitsudomi T Morita S Yatabe Y Negoro S Okamoto I Tsurutani J . Gefitinib versus cisplatin plus docetaxel in patients with non-small-cell lung cancer harbouring mutations of the epidermal growth factor receptor (WJTOG3405): an open label, randomised phase 3 trial. Lancet Oncol. (2010) 11(2):121–8. doi: 10.1016/S1470-2045(09)70364-X 20022809

[3] Thress K Paweletz C Felip E Cho B Stetson D Dougherty B . Acquired EGFR C797S mutation mediates resistance to AZD9291 in non-small cell lung cancer harboring EGFR T790M. Nat Med. (2015) 21(6):560–2. doi: 10.1038/nm.3854 PMC4771182 25939061

[4] Lin CC Shih JY Yu CJ Ho CC Liao WY Lee JH . Outcomes in patients with non-small-cell lung cancer and acquired Thr790Met mutation treated with osimertinib: a genomic study. Lancet Respir Med. (2018) 6:107–16. doi: 10.1016/S2213-2600(17)30480-0 29249325

[5] Oxnard GR Hu Y Mileham KF Husain H Costa DB Tracy P . Assessment of resistance mechanisms and clinical implications in patients with EGFR T790M-positive lung cancer and acquired resistance to osimertinib. JAMA Oncol. (2018) 4:1527–34. doi: 10.1001/jamaoncol.2018.2969 PMC6240476 30073261

[6] Yang Z Yang N Ou Q Xiang Y Jiang T Wu X . Investigating novel resistance mechanisms to third-generation EGFR tyrosine kinase inhibitor osimertinib in non-small cell lung cancer patients. Clin Cancer research: an Off J Am Assoc Cancer Res. (2018) 24:3097–107. doi: 10.1158/1078-0432.CCR-17-2310 29506987

[7] Niederst M Hu H Mulvey H Lockerman E Garcia A Piotrowska Z . The allelic context of the C797S mutation acquired upon treatment with third-generation EGFR inhibitors impacts sensitivity to subsequent treatment strategies. Clin Cancer Res. (2015) 21(17):3924–33. doi: 10.1158/1078-0432.CCR-15-0560 PMC4587765 25964297

[8] Wang Z Yang J Huang J Ye J Zhang X Tu H . Adenocarcinoma harboring EGFR T790M and in trans C797S responds to combination therapy of first- and third-generation EGFR TKIs and shifts allelic configuration at resistance. Lung. (2017) 12:1723–7. doi: 10.1016/j.jtho.2017.06.017 28662863

[9] Hidaka N Iwama E Kubo N Harada T Miyawaki K Tanaka K . Most T790M mutations are present on the same EGFR allele as activating mutations in patients with non-small cell lung cancer. Lung Cancer. (2017) 108:75–82. doi: 10.1016/j.lungcan.2017.02.019 28625653

[10] Ettinger D Wood D Aisner D Akerley W Bauman J Bharat A . Non-small cell lung cancer, version 3.2022, NCCN Clinical Practice Guidelines in Oncology. J Natl Compr Canc Netw. (2022) 20(5):497–530. doi: 10.6004/jnccn.2022.0025 35545176

[11] Lu S Wu L Jian H Chen Y Wang Q Fang J . Sintilimab plus bevacizumab biosimilar IBI305 and chemotherapy for patients with EGFR-mutated non-squamous non-small-cell lung cancer who progressed on EGFR tyrosine-kinase inhibitor therapy (ORIENT-31): first interim results from a randomised, double-blind, multicentre, phase 3 trial. Lancet Oncol. (2022) 23:1167–79. doi: 10.1016/S1470-2045(22)00382-5 35908558

[12] Bertoli E De Carlo E Del Conte A Stanzione B Revelant A Fassetta K . Acquired resistance to osimertinib in EGFR-mutated non-small cell lung cancer: how do we overcome it? Int J Mol Sci. (2022) 23. doi: 10.3390/ijms23136936 PMC9266773 35805940

[13] Ferro A Marinato GM Cristiana M Marino M Pasello G Guarneri V . Primary and acquired resistance to first-line Osimertinib to improve the outcome of EGFR-mutated advanced non-small cell lung cancer patients: the challenge is open for new therapeutic strategies. Crit Rev oncology/hematology. (2024) 104295. doi: 10.1016/j.critrevonc.2024.104295 38382773

[14] Yang Y Xu H Ma L Yang L Yang G Zhang S . Possibility of brigatinib-based therapy, or chemotherapy plus anti-angiogenic treatment after resistance of osimertinib harboring EGFR T790M-cis-C797S mutations in lung adenocarcinoma patients. Cancer Med. (2021) 10:8328–37. doi: 10.1002/cam4.4336 PMC8633234 34612594

[15] Wang Y Yang N Zhang Y Li L Han R Zhu M . Effective treatment of lung adenocarcinoma harboring EGFR-activating mutation, T790M, and cis-C797S triple mutations by brigatinib and cetuximab combination therapy. J Thorac oncology: Off Publ Int Assoc Study Lung Cancer. (2020) 15:1369–75. doi: 10.1016/j.jtho.2020.04.014 32353596

[16] Maity S Pai KSR Nayak Y . Advances in targeting EGFR allosteric site as anti-NSCLC therapy to overcome the drug resistance. Pharmacol reports: PR. (2020) 72:799–813. doi: 10.1007/s43440-020-00131-0 32666476 PMC7381467

[17] Passaro A Wang J Wang Y Lee SH Melosky B Shih JY . Amivantamab plus chemotherapy with and without lazertinib in EGFR-mutant advanced NSCLC after disease progression on osimertinib: primary results from the phase III MARIPOSA-2 study. Ann oncology: Off J Eur Soc Med Oncol. (2024) 35:77–90. doi: 10.1016/j.annonc.2023.11.012 37879444

[18] Yu HA Goto Y Hayashi H Felip E Chih-Hsin Yang J Reck M . HERTHENA-lung01, a phase II trial of patritumab deruxtecan (HER3-DXd) in epidermal growth factor receptor-mutated non-small-cell lung cancer after epidermal growth factor receptor tyrosine kinase inhibitor therapy and platinum-based chemotherapy. J Clin oncology: Off J Am Soc Clin Oncol. (2023) 41:5363–75. doi: 10.1200/JCO.23.01476 PMC10713116 37689979

[19] Reck M Mok TSK Nishio M Jotte RM Cappuzzo F Orlandi F . Atezolizumab plus bevacizumab and chemotherapy in non-small-cell lung cancer (IMpower150): key subgroup analyses of patients with EGFR mutations or baseline liver metastases in a randomised, open-label phase 3 trial. Lancet Respir Med. (2019) 7:387–401. doi: 10.1016/S2213-2600(19)30084-0 30922878

[20] Nogami N Barlesi F Socinski MA Reck M Thomas CA Cappuzzo F . IMpower150 final exploratory analyses for atezolizumab plus bevacizumab and chemotherapy in key NSCLC patient subgroups with EGFR mutations or metastases in the liver or brain. J Thorac oncology: Off Publ Int Assoc Study Lung Cancer. (2022) 17:309–23. doi: 10.1016/j.jtho.2021.09.014 34626838

[21] Hastings K Yu HA Wei W Sanchez-Vega F DeVeaux M Choi J . EGFR mutation subtypes and response to immune checkpoint blockade treatment in non-small-cell lung cancer. Ann oncology: Off J Eur Soc Med Oncol. (2019) 30:1311–20. doi: 10.1093/annonc/mdz141 PMC6683857 31086949

[22] Isomoto K Haratani K Hayashi H Shimizu S Tomida S Niwa T . Impact of EGFR-TKI treatment on the tumor immune microenvironment in EGFR mutation-positive non-small cell lung cancer. Clin Cancer research: an Off J Am Assoc Cancer Res. (2020) 26:2037–46. doi: 10.1158/1078-0432.CCR-19-2027 31937613

[23] Haratani K Hayashi H Tanaka T Kaneda H Togashi Y Sakai K . Tumor immune microenvironment and nivolumab efficacy in EGFR mutation-positive non-small-cell lung cancer based on T790M status after disease progression during EGFR-TKI treatment. Ann oncology: Off J Eur Soc Med Oncol. (2017) 28:1532–9. doi: 10.1093/annonc/mdx183 28407039

[24] Peng S Wang R Zhang X Ma Y Zhong L Li K . EGFR-TKI resistance promotes immune escape in lung cancer *via* increased PD-L1 expression. Mol Cancer. (2019) 18:165. doi: 10.1186/s12943-019-1073-4 31747941 PMC6864970

[25] Chen DS Mellman I . Oncology meets immunology: the cancer-immunity cycle. Immunity. (2013) 39:1–10. doi: 10.1016/j.immuni.2013.07.012 23890059

[26] Voron T Colussi O Marcheteau E Pernot S Nizard M Pointet AL . VEGF-A modulates expression of inhibitory checkpoints on CD8+ T cells in tumors. J Exp Med. (2015) 212:139–48. doi: 10.1084/jem.20140559 PMC4322048 25601652

[27] Jiang T Wang P Zhang J Zhao Y Zhou J Fan Y . Toripalimab plus chemotherapy as second-line treatment in previously EGFR-TKI treated patients with EGFR-mutant-advanced NSCLC: a multicenter phase-II trial. Signal transduction targeted Ther. (2021) 6:355. doi: 10.1038/s41392-021-00751-9 PMC8517012 34650034

[28] Hegde PS Wallin JJ Mancao C . Predictive markers of anti-VEGF and emerging role of angiogenesis inhibitors as immunotherapeutics. Semin Cancer Biol. (2018) 52:117–24. doi: 10.1016/j.semcancer.2017.12.002 29229461

[29] Chen DS Hurwitz H . Combinations of bevacizumab with cancer immunotherapy. Cancer J (Sudbury Mass). (2018) 24:193–204. doi: 10.1097/PPO.0000000000000327 30119083

[30] Bancroft CC Chen Z Yeh J Sunwoo JB Yeh NT Jackson S . Effects of pharmacologic antagonists of epidermal growth factor receptor, PI3K and MEK signal kinases on NF-kappaB and AP-1 activation and IL-8 and VEGF expression in human head and neck squamous cell carcinoma lines. Int J Cancer. (2002) 99:538–48. doi: 10.1002/ijc.10398 11992543

[31] Hung MS Chen IC Lin PY Lung JH Li YC Lin YC . Epidermal growth factor receptor mutation enhances expression of vascular endothelial growth factor in lung cancer. Oncol Lett. (2016) 12:4598–604. doi: 10.3892/ol.2016.5287 PMC5228119 28101216

